# COVID-19-mandated social restrictions unveil the impact of social time pressure on sleep and body clock

**DOI:** 10.1038/s41598-020-79299-7

**Published:** 2020-12-17

**Authors:** Maria Korman, Vadim Tkachev, Cátia Reis, Yoko Komada, Shingo Kitamura, Denis Gubin, Vinod Kumar, Till Roenneberg

**Affiliations:** 1 Department of Occupational Therapy, Faculty of Health Sciences, Ariel University, Ariel, Israel; 2 Unaffiliated, Rehovot, Israel; 3 ISAMB, Faculdade de Medicina, Universidade de Lisboa, Lisbon, Portugal; 4 Faculdade de Medicina de Lisboa, Instituto de Medicina Molecular João Lobo Antunes, Universidade de Lisboa, Lisbon, Portugal; 5 CENC - Centro de Medicina de Sono, Lisbon, Portugal; 6 Liberal Arts, Meiji Pharmaceutical University, Tokyo, Japan; 7 Department of Sleep-Wake Disorders, National Institute of Mental Health, National Center of Neurology and Psychiatry, Tokyo, Japan; 8 Department of Biology, Medical University, Tyumen, Russia; 9 Tyumen Cardiology Research Center, Tomsk National Research Medical Center, Russian Academy of Science, Tomsk, Russia; 10 Department of Zoology, University of Delhi, Delhi, India; 11 Institute and Polyclinic for Occupational-, Social- and Environmental Medicine, LMU Munich, Munich, Germany; 12 Chronsulting UG, Dietersburg, Germany

**Keywords:** Circadian rhythms and sleep, Circadian regulation, Epidemiology

## Abstract

In humans, sleep regulation is tightly linked to social times that assign local time to events, such as school, work, or meals. The impact of these social times, collectively—social time pressure, on sleep has been studied epidemiologically via quantification of the discrepancy between sleep times on workdays and those on work-free days. This discrepancy is known as the social jetlag (SJL). COVID-19-mandated social restrictions (SR) constituted a global intervention by affecting social times worldwide. We launched a Global Chrono Corona Survey (GCCS) that queried sleep–wake times before and during SR (*preSR* and *inSR*). 11,431 adults from 40 countries responded between April 4 and May 6, 2020. The final sample consisted of 7517 respondents (68.2% females), who had been 32.7 ± 9.1 (mean ± sd) days under SR. SR led to robust changes: mid-sleep time on workdays and free days was delayed by 50 and 22 min, respectively; sleep duration increased on workdays by 26 min but shortened by 9 min on free days; SJL decreased by ~ 30 min. On workdays *inSR*, sleep–wake times in most people approached those of their *preSR* free days. Changes in sleep duration and SJL correlated with *inSR*-use of alarm clocks and were larger in young adults. The data indicate a massive sleep deficit under pre-pandemic social time pressure, provide insights to the actual sleep need of different age-groups and suggest that tolerable SJL is about 20 min. Relaxed social time pressure promotes more sleep, smaller SJL and reduced use of alarm clocks.

## Introduction

Circadian clocks are fundamental biological functions that exist even in single-cell cyanobacteria, with fossils dating up to 3.5 billion years^1^. Circadian clocks generate self-sustained 24-h rhythms endogenously^2^ that synchronize actively to the light–dark cycle (entrainment), with a specific phase relationship (phase of entrainment^3^). The endogenous circadian clocks therefore help tune the organisms’ physiology with the predictable cyclic environment generated by the alternation of day and night, of light and darkness, and of cold and warmth, all of which may cause time-of-day specific resources or enemies. The role of social stimuli, such as school, work, meetings or meals, in regulation of human circadian rhythms is not fully understood^4^.

The circadian clock in humans (here referred to as the body clock) is most apparent at the behavioral level; for example, by controlling the sleep–wake cycle. For more than 80 years, the body clock has been experimentally studied by isolating humans in caves^5^ or in the laboratory^6^ from the cyclic cues of the daily environment (“zeitgebers”) or by scheduling them to non-24-h regimes^7^. The body clock has also been studied epidemiologically, mostly with the help of questionnaires about sleep times^8–10^. From these studies, the concept of “chronotypes” (e.g., “owls” and “larks”) has emerged^11^. This concept helps to describe the phenotypic variance in how individual clocks entrain to the light–dark cycle, resulting in different phases of entrainment. The distribution of phases (chronotypes) has become wider and later during industrialization due to weakening the zeitgeber: humans are exposed to less light during the day (by living predominantly inside) and less darkness during the night (by using artificial light).

Work schedules have not been adapted to the later body clocks, leading to a discrepancy between internal, circadian, time and external, social, time constraints. This discrepancy has been coined “social jetlag” (SJL)^12^, and has been associated with health^13–20^, well-being^21^ and performance deficits^22–26^. SJL is similar to living in the “wrong time zone”, but in contrast to jet lag caused by travel, SJL is chronic since circadian clocks cannot resynchronize to a new zeitgeber under conditions of perpetual shifting between different social times during workdays and during work-free days. Typically, SJL is greater in late chronotypes because their sleep episode is shortened at both the beginning and end: the late opening of the circadian sleep window^27^ causes late sleep onset and, on workdays, the early awakening is artificially induced in order to comply with the morning-oriented social times (e.g., school/work start times). The mismatch between the temporal window provided by the body clock for optimal sleep and that “provided” by social times, collectively—social time pressure (STP), has led to an almost ubiquitous use of alarm clocks in modern societies. Needing an alarm clock to wake up simply means that the sleep episode would have ended later if there were no alarm clock. Regularly shortening sleep results in sleep deficits and is associated with prolonged sleep inertia and health and performance deficits^28, 29^.

Different chronotypes change their synchronization differentially when exposed to the weak zeitgebers. These changes are reversible as was shown by Ken Wright’s camping studies^30^; when urbanized people are exposed to strong zeitgebers (e.g., by going camping for one week), the late chronotypes respond with an especially large clock advance, suggesting that the circadian changes experienced in industrialization can be reversed. While camping versus urban conditions allowed investigating the impact of zeitgeber strength on the body clock, the transition to pandemic-related social restrictions (SR) offered a unique possibility to investigate the impact of STP on the body clock in situ.

Previously, circadian research in humans was predominantly conducted in laboratory conditions shielding participants from the natural zeitgeber by eliminating knowledge about natural daylight. Covid-19-mandated restrictions provided shielding of circadian clocks from STP due to work-from-home (canceling the need to spend time on commuting between home and work), more flexible home office hours and social distancing. These conditions offered a unique opportunity to quantify the impact of relaxed STP in a pre-post intervention within-subject design. SR also can dramatically decrease the time spent outdoors, yet knowledge about the natural zeitgeber is still available. Here, we investigated the consequences of the COVID-19 mandated SR during spring 2020 with an overarching aim to better understand the interaction between STP, sleep and body clock.

The internet-based Global Chrono Corona Survey (GCCS) asked participants about their sleep times on work and work-free days, use of alarm clocks and the time spent outdoors in daylight both before and during social restrictions. 11,431 adults from 40 countries responded between April 4 and May 6, 2020. The final sample consisted of 7517 respondents (68.2% females), who had been 32.7 ± 9.1 (mean ± sd) days under SR (presumably allowing full adaptation to new social times). Participants could choose a preferred language. Respondents were asked to answer questions about daily behavior and lifestyle in separate sections, each pertaining to before social restrictions (preSocialRestriction, *preSR*) and during the restrictions (inSocialRestriction, *inSR*). 80% of respondents worked or studied both *preSR* and *inSR* (66% from home). Full sociodemographic details are presented in Supplementary Material (SI-Table S1).

The following daily behaviors were calculated for each individual: (i) Mean sleep duration across the week (SD_week_) was calculated as weighted average of the sleep duration on workdays (SD_W_, assuming 5 workdays) and work-free days (SD_F_), [SD_week_ = (5 × SD_W_ + 2 × SD_F_)/7]. (ii) Chronotypes (MSF_sc_), quantifying individual sleep timing as an indicator for phase of entrainment^11^. MSF_sc_ is based on the mid-point of sleep on free days (MSF) and corrected for sleep deficit accumulated on workdays. The mid-point of sleep was also calculated for workdays (MSW). (iii) As an assessment for circadian misalignment, social jet-lag (SJL) was calculated as the difference between MSF and MSW. To quantify the magnitude of changes in daily behavior we calculated individual restriction-induced Deltas (**∆**) as [*inSR*-*preSR*] for each parameter. We expected to obtain shorter SD_week_, earlier MSF_sc_ and smaller SJL *preSR* compared to *inSR*. Furthermore, we hypothesized that the changes in daily parameters will be age-dependent, with larger changes in young participants. We likewise expected a lower frequency of alarm clock *inSR* and that the participants who stopped using the alarm clock will show larger changes in sleep–wake parameters.

## Results

### Sleep duration (SD_week_), midsleep timing (MSF_sc_) and social jetlag (SJL)

Social restrictions lead to robust shifts in daily behavior and in exposure to daylight. The average outdoor daylight exposure showed a twofold decrease *inSR*: from 2 h 21 min ± 102 min to 1 h 8 min ± 75 min (Z =  − 63.47, p < 0.001, r = 0.73; Wilcoxon Signed Ranks test).

**Figure 1:**
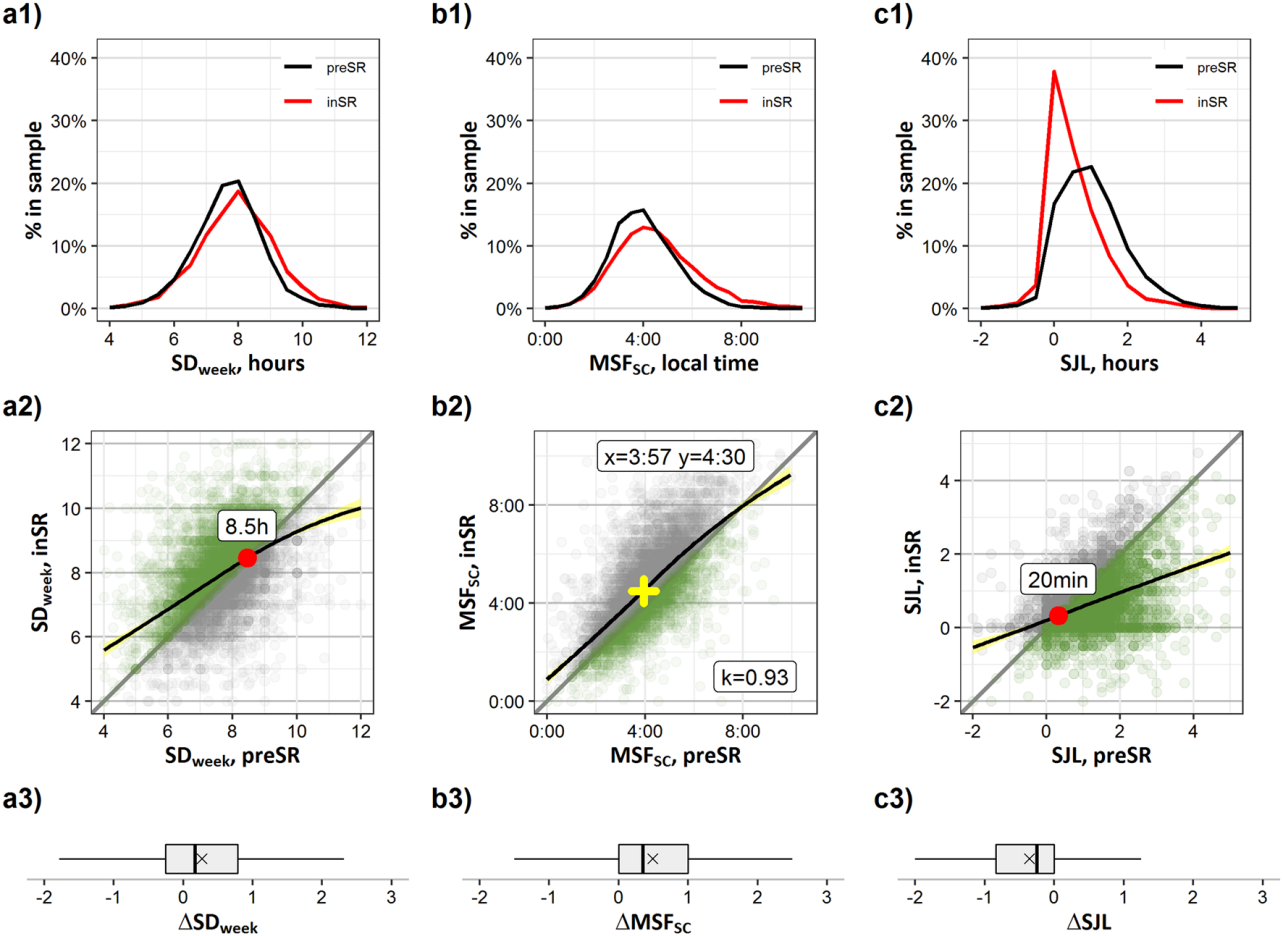
Social restriction-induced changes (*preSR *→ *inSR*) in sleep–wake behavior in the general sample. (**a1**–**c1**) Distributions of SD_week_, MSF_sc_, and SJL *preSR* (black line) and *inSR* (red line), percent from group total. (**a2**–**c2**) Scatterplots of sleep duration, SD_week_ (hours), corrected mid-sleep time on free days, MSF_sc_ (chronotype, local time), and social jetlag, SJL (hours), *preSR* (x axis) vs. *inSR* (y axis). Each dot represents an individual participant, overlapping dots are coded by color intensity. Diagonal line designates no restriction-induced change in parameter. Green—increase/advance, grey—decrease/delay. Black line—LOESS regression lines illustrate the relationship between the parameter values *preSR* (x-axis) and the smoothed parameter values *inSR* (y-axis), pointwise 95% confidence intervals are visualized by bands shaded in yellow. Red dot—intersection point between the diagonal and the LOESS line. Yellow cross coordinates—means of MSF_sc_ *preSR* and *inSR*; k—slope of the tangent for the LOESS regression line at the point of mean MSF_sc_ *preSR.* (**a3**–**c3**) Boxplots of individual differences (∆, hours) in SD_week_, MSF_sc_ and SJL. Positive values—increase in SD_week_, delay in MSF_sc_. Negative values—decrease in SJL. Whiskers—max and min values, box borders—75th and 25th percentiles, line through the box—median, ×marker—mean.

During SR, the individual SD_week_ increased on average by 15 min (∆SD_week_, Z =  − 21.297, p < 0.001, r = 0.25; Fig. 1, panels a1–a3). This shift reflects both a robust increase in the sleep duration on workdays (∆SD_**W**_, + 26 min, + 5.84%; Z =  − 28.159, p < 0.001, r = 0.36) and a small but consistent decrease in the sleep duration on work-free days (∆SD_**F**_, − 9 min, − 1.93%; Z =  − 11.430, p < 0.001, r = 0.13). More participants reported no changes (42.8%) in sleep quality than those who had negative (i.e., worse sleep quality, 34.2%) or positive (23.0%) changes. Distributions and individual shifts of the SD_W_, SD_F_, MSW and MSF, are shown in Supplementary Information (SI-Fig. S1).

Moreover, *inSR*, the individual MSF_sc_ (chronotypes) delayed, on average, by 34 min (Z =  − 42.363, p < 0.001, r = 0.49; Fig. 1, panels b1–b3). The most prominent delay in sleep timing happened during workdays (∆MSW, 50 min) (Z =  − 49.051, p < 0.001, r = 0.63). During work-free days, a significant delay in the mid-point of sleep was also observed (∆MSF, 22 min) (Z =  − 32.070, p < 0.001, r = 0.37). SJL decreased on average by 29 min (Z =  − 39.386, p < 0.001, r = 0.5; Fig. 1, panels c1–c3), indicating a robust alignment of sleep times between work- and work-free days during the *inSR* period.

Non-parametric LOESS regressions show the smoothed relationships between the parameters of daily behavior *preSR* and *inSR* (Fig. 1, panels a2–c2). People who sleep shorter than ~ 8.5 h *preSR* tend to lengthen their sleep under the relaxed social clock (*inSR*), while those who slept even longer than ~ 8.5 h *preSR* tend to sleep shorter, and only those who slept around 8.5 h on average did not change (Fig. 1, intersection indicated by red dot, panel a2). SJL shows a crossing point value around ~ 20 min (Fig. 3, red dot, panel c2); those with SJL > 20 min tend to reduce it, while those who had SJL < 20 min *preSR* (only 18% of the sample) tend to have small or no change in SJL *inSR*. The fact that people suffering from ~ 20 min of SJL do not change their habits to reduce SJL suggests this amount of circadian misalignment can be tolerated. Note that some participants (17% of the sample) increased SJL (Fig. 1, panel c2 grey dots), an evidence for an increase in social time pressure *inSR* for these individuals. The LOESS regression for MSF_sc_ has a slope (k) close to 1, showing an almost constant delay across all chronotypes. Its variance reflects the known wide range of chronotypes in industrialized populations, but the distribution is delayed (e.g., shifted above the diagonal) as a whole between *preSR* and *inSR* (similar to age changes^31^) (Fig. 1, panel b2; 95% of the sample is to the left of the intersection point).

The deviation of the SD_week_ and SJL slopes of the LOESS curves from the diagonal suggested that the response to relaxing social constraints depended on the initial, pre-pandemic, sleep duration and SJL: the more under-slept and SJL-misaligned people were *preSR*, the more they increased sleep duration or decreased SJL *inSR*. We quantified the *preSR*-*inSR* differences (∆) using Spearman’s rank correlations against the *preSR*-value. Indeed, the correlation of SD_week_ *preSR* and ∆SD_week_ was negative (ρ =  − 0.302, p < 0.001). Since respondents were, on average, 3–5 weeks into the SR period, we assume that the gains in SD_week_ presumably reflect individual sleep-need rather than a compensating of the previous sleep debt. Similarly, there was a negative correlation between the SJL level before the restriction and ∆SJL (Spearman’s ρ =  − 0.593, p < 0.001), i.e., ∆SJL was more robust in individuals who initially suffered from stronger circadian misalignment. As to be expected from the results described above, ∆MSF_**sc**_ was independent of the *preSR*-MSF_**sc**_ times (Spearman’s ρ =  − 0.006, p = 0.632).

Changes in sleep duration and timing were driven by delays in sleep onset (time of falling asleep) and sleep offset (awakening) times, during both workdays and free days *inSR*, see Supplementary Information (SI-Fig. S2). Note that the delay in sleep offset times on workdays (64 min) was on average twice as large as the delay in onset times (37 min). Thus, longer sleep duration *inSR* is subserved primarily by postponing the time of awakening on workdays.

### Age related changes

There were no differences between the sexes in ∆SD_week_ and minor but significant differences in ∆MSF_sc_ (7.8 min) and ∆SJL (6.7 min), with negligible effect sizes (r_g_ < 0.1), see Supplementary Information (SI-Fig. S3). All shifts in daily behavior (all deltas) significantly correlated with age overall (Table 1; note negligible ρ for ∆SD_F_).

**Table 1:** Spearman’s correlations between responders’ age and deltas in daily behavior parameters.

	∆SD_week_	∆SD_W_	∆SD_F_	∆MSF_sc_	∆MSW	∆MSF	∆SJL
Age	− 0.115*	− 0.142*	0.061*	− 0.204*	− 0.290*	− 0.131*	0.267*

Values—Spearman’s ρ.

*p < 0.001.

**Figure 2:**
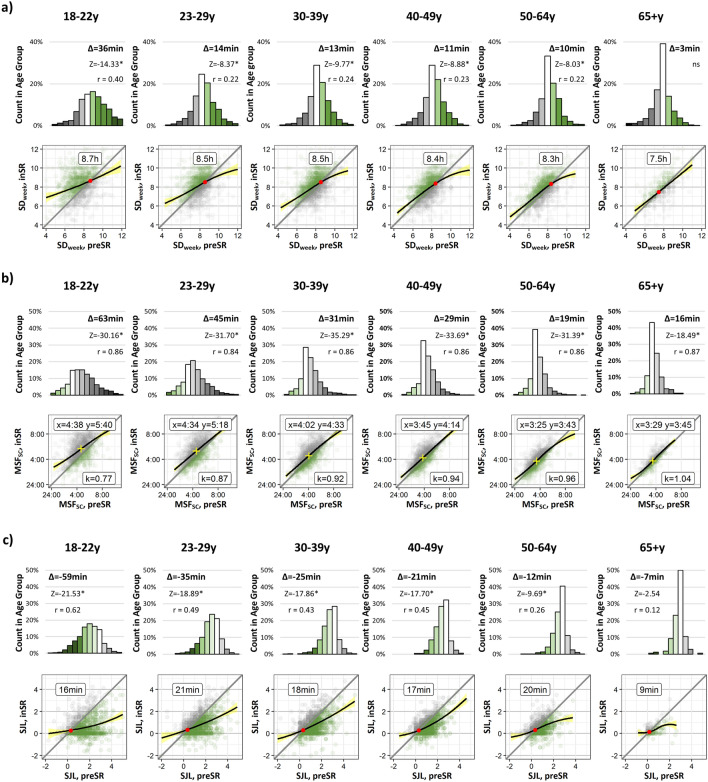
Social restriction-induced changes (*preSR *→ *inSR*) in sleep–wake behavior by age-group (18–22y, 23–29y, 30–39y, 40–49y, 50–64y, and 65 + y). Upper panels—distributions of (**a**) delta of sleep duration, ∆SD_week_, (**b**) delta of corrected mid-sleep time on free days, ∆MSF_sc_ (chronotype), (**c**) delta of social jetlag, ∆SJL. The magnitude of shifts is represented in 0.5 h color-coded bins, with white bars representing no change, green bar—increase in SD_week_, advance in MSF_sc_ and decrease in SJL, grey bars—decrease in SD_week_, delay in MSF_sc_ and increase in SJL. Mean ∆ in minutes and Z statistic of the post-hoc Wilcoxon Signed Ranks two-tailed tests for each parameter are shown above the bars. *p < 0.001, *r* effect size, *ns* non-significant. Lower panels—Scatterplots of individual shifts in (**a**) SD_week_ (h), (**b**) MSF_sc_ (chronotype, local time), and (**c**) SJL (h), *preSR* (x axis) vs. *inSR* (y axis). Notations—as in Fig. 1a2–c2.

To further explore these age-effects, separate post-hoc analyses were performed using six arbitrary age groups distributed, so that each had approximately the same sample size: 18–22y (N = 1225), 23–29y (N = 1340), 30–39y (N = 1660), 40–49y (N = 1515), 50–64y (N = 1312), and a smaller senior group, 65 + y (N = 456) (Fig. 2; see socio-demographic details of the age groups in SI-Table S2). The overall magnitude of the shifts was estimated via the mean values of deltas (given in minutes together with the Z-scores of the Wilcoxon Matched-Pairs two-tailed tests above each distribution panel). The magnitude of the respective changes decreased consistently with age and did not even reach significance for ∆SD_week_ in the 65 + y age group. For MSF_sc_, a chronotype-independent shift (i.e., slope close to 1), observed for the total study population (Fig. 1b2), exists only in the middle-age and older age groups. In young participants, 18–22y and 25–29y, the early-type participants (i.e., MSF_sc_ < 2:00) delayed relatively more than late types (i.e., MSF_sc_ > 5:00) (Fig. 2b, k values). Except for the senior group (65 + y), the crossing points for SJL in the individual groups were similar to each other (Fig. 1c2). The age dependency of the magnitude in shifts of SD_week_, MSF_sc_ and of the SJL can be visualized in several aspects: (i) the mean values at the top of each panel demonstrate the progressive decrease in ∆SD_week_ and ∆SJL values with older age (ii) the proportion of the group without change—height of white central bars in the ∆-distributions, is increasing with age; (iii) the kurtosis of the ∆SD_week_, ∆MSF_sc_, and ∆SJL distributions in decreasing with age. Changes in sleep onset and sleep offset times *inSR* were larger in young adults and decreased with age (SM-Fig. S4, separate panels for workdays and free days).

### Use of alarm clock and working from home

In modern societies, compliance with social times is ubiquitously achieved by using an alarm clock. Among working/studying participants (N = 6012, 80% of the total sample), *preSR,* 84% usually used alarm clocks during workdays and 12% worked from home, while inSR only 56% of working participants woke up with alarm clock on workdays and 82% worked from home. Before the pandemic, alarm clock users (N = 5060) were—on average—later chronotypes, had shorter sleep on workdays and longer sleep on work-free days than participants who did not use alarm clocks (N = 952) and consequently suffered from more SJL (Mann–Whitney tests: MSF_sc_: + 35 min, Z =  − 15.44, p < 0.001, r_g_ =  − 0.31; SJL: + 46 min, Z =  − 26.33, p < 0.001, r_g_ =  − 0.54; SD_W_: − 19 min, Z =  − 8.48, p < 0.001, r_g_ = 0.17; SD_F_: + 28 min; Z =  − 11.18, p < 0.001, r_g_ =  − 0.22). Alarm clock use *preSR* was more prevalent among adults < 50 years, compared to older adults (18–22y—89%, 23–29y—86%, 30–39y—80%, 40–49y—81%, 50–64y—66%, 65 + y—23%). The low prevalence of alarm clock use in 65 + y group presumably related to retirement since only 48% were employed *preSR*. To evaluate this assumption, we calculated the alarm-use/employment ratio (see SI-Table S2, Ratio (Alarm clock usage/Employed)). This ratio declines with age *preSR,* suggesting that employment status is not a sole factor in alarm use on workdays.

**Figure 3:**
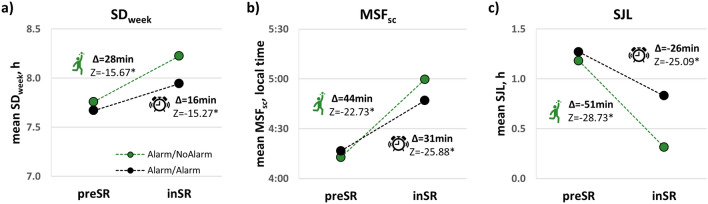
The contribution of alarm clock use on workdays to changes in daily behavior in participants who work from home *inSR* and used alarm clock *preSR*: Alarm/NoAlarm (green markers) and Alarm/Alarm (black markers) groups. Mean values of individual (**a**) SD_week_ (h), (**b**) MSF_sc_ (chronotype, local time), and (**c**) SJL (h) parameters *preSR* and *inSR*. Mean ∆ in minutes and Wilcoxon Matched-Pairs two-tailed tests Z statistic for each parameter are shown on the graphs, *p < 0.001.

To assess the impact of the relaxed social time pressure (STP) and alarm-clock use on changes in daily behavior, we selected a group of participants who worked/studied both *preSR* and *inSR*, used an alarm clock on workdays *preSR,* and worked/studied from home *inSR*. This group (N = 4135) was then subdivided into those who stopped using an alarm clock *inSR* (Alarm/NoAlarm; N = 1539 [37%]) and those who continued to use alarm clock inSR (Alarm/Alarm; N = 2596 [63%]). The two groups were similar in age and sex composition (SI-Table S3). While both groups showed robust changes in sleep duration, mid-sleep time, and SJL (Fig. 3), magnitudes of changes were significantly larger in the Alarm/NoAlarm group than in the Alarm/Alarm group (Mann–Whitney tests; ∆SD_week_: Z =  − 5.957, p < 0.001, r_g_ = 0.11; ∆MSF_sc_: Z =  − 7.101, p < 0.001, r_g_ = 0.13; ∆SJL: Z = − 15.227, p < 0.001, r_g_ = 0.28; complementary LOESS regressions and box plots of individual differences in SI-Fig. S4).

The groups did not differ in sleep onset and sleep offset timings on workdays *preSR* (Table 2). *InSR*, both groups fell asleep and woke up later, but these changes were significantly larger in the Alarm/NoAlarm group. Thus, the differences in SD_week_, MSF_sc_ and SJL between the groups are primarily related to alarm-independent awakening on workdays in the Alarm/NoAlarm group. Mann–Whitney tests were also used to compare the people who worked from home (4977) to those that worked not from home and presumably had stricter schedules (1126), *inSR*. All deltas (∆SD_week_, ∆MSF_sc_ and ∆SJL) were larger in the “worked from home” subgroup (SI-Table S4).

**Table 2:** Timings (mean ± sd, local time) of falling asleep and waking-up on workdays *preSR* and *inSR* by group, Alarm/NoAlarm and Alarm/Alarm.

	Sleep onset time				Sleep offset time			
	*preSR*	*inSR*	Δ	Z	*preSR*	*inSR*	Δ	Z
Alarm/NoAlarm	23:42 ± 1:14	0:39 ± 1:52	0:56 ± 1:29	9.58** r_g_ = 0.18	7:08 ± 1:09	8:49 ± 1:52	1:41 ± 1:41	15.42** r_g_ = 0.26
Alarm/Alarm	23:49 ± 1:10	0:23 ± 1:34	0:34 ± 1:09		7:07 ± 1:04	8:07 ± 1:34	1:00 ± 1:13	

Changes [*inSR- preSR*] are designated by Δ. Mann–Whitney Z scores indicate the differences in Δ values between the groups, *r*_*g*_ effect size.

**p < 0.001.

## Discussion

The SR issued as a response to the Covid-19 pandemic constituted a huge circadian experiment. Weaker STP due to home office, no commute, and possibly less stringent workhours most frequently accompanies the implemented SR. Before SR, many people suffered from a mismatch between circadian times (e.g., when we best sleep) and social times (e.g., when we go to work, school). This misalignment (social jetlag, SJL) is associated with health and performance deficits and becomes apparent in the usage of alarm clocks that shorten our biological sleep program due to social time pressure (STP). More than 80% of the working population uses alarm clocks on workdays^32^, which was replicated here for the *preSR* ‘normality’ (84% use an alarm clock).

The two conditions (*preSR, inSR*) allowed computing within-subject changes in variables, such as mid-sleep times, sleep duration or SJL. The most important outcome of the GCCS study for circadian/sleep and public health research is the obtained robust gain in sleep duration *inSR* compared to *preSR,* accompanied by a substantial reduction of SJL. These benefits were achieved without major negative changes in sleep quality, in line with the parallel findings by Leone et al.^33^. Our findings are also in line with the results of several smaller scale studies of the impact of COVID-19 mandated SR during spring 2020 on sleep and circadian behavior^34, 35^. The GCCS sample is large-scale and represents adults from all age groups. In conjunction with a within-individual analysis these sample characteristics provided a chance to quantify in great detail sleep-related variables (duration, timing and SJL) and determine actual sleep-need more accurately via modelling.

The *preSR-inSR* slopes of regression line in sleep duration and SJL deviate substantially from 1 (Fig. 1, row 2 and second row of panels in Fig. 2a,c). One interpretation of this “rubber-band” effect is that the further apart the social and the biological time-zone (the greater the circadian strain) the further individuals “travel” (relax) when STP weakens. The difference between these allegorical time-zones strongly correlates with SJL, and the larger its extent the greater the change when STP weakens. Greater SJL is associated with shorter average sleep duration^11^, consequently, the shorter people sleep *preSR*, the more they extend their sleep *inSR*.

Traditionally, differences in sleep duration between workdays and free days were used to describe longer sleep duration on free days as compensatory for the sleep debt accumulated over the workweek, especially in adolescents and young adults^36^. However, our current results show that sleep timing and duration on free days primarily reflect the actual individual sleep need and not a compensation for sleep debt accumulated on workdays: after full adaptation to the relaxed STP, sleep duration on workdays is lengthened by about 6%, while on free days sleep duration is shortened by only 2%.

The large number of participants in our GCCS database allowed us to look at different age groups, and results show age-dependencies in all variables (Table 1). Adolescents are the latest chronotypes among in any population^31, 37, 38^ (SI-Table S2, mean MSFsc by age group) and therefore are exposed to the most rigorous STP leading to the largest sleep deprivation and SJL^17^. As to be expected, this age group shows the strongest responses to weaker STP *inSR*^39^. The 65 + y age group is exposed to the weakest STP under *preSR* conditions (only 48% are employed compared to 89–99% in other age groups, see SI-Table S2) and consequently shows only small changes in response to social restrictions.

While the 18–22y age group increases average sleep duration across the week (SD_week_) by on average 36 min, the 65 + y age group gains 3 min; while the youngest age group in our population reduces SJL by almost one hour, the 65 + y participants get a relief of 7 min. Not mutually exclusive is the possibility that reduced sensitivity to environmental zeitgebers (primarily, light) could contribute to reduced circadian response to social restrictions induced changes in later life^40^. Our data suggests that age-dependent disparities in sleep duration gains are also likely to be mediated by differences in both the employment-related STP and alarm-use in different age groups (SI-Table S2).

Regression lines for both sleep duration and SJL produce intersects with the 1:1 diagonal (Fig. 1a2,c2, red dots): the intersect for sleep duration in the entire cohort is ~ 8.5 h and that for SJL 20 min. We propose that these intersects might be predictors for *sleep need* and for the *tolerance for SJL*, respectively. When segregated into age groups, the intersects indicate a higher sleep need for younger people that steadily decreases with age (Fig. 2a). This is in line with previously published data^17^ and roughly corresponds to the average free-day sleep duration in the respective age groups. The intersect for the SJL scatter plots is close to the 20-min tolerance in all age groups except for the elderly. If one considers this limit of tolerance to distinguish between affected and non-affected people, then the prevalence of SJL on population level would be 88%^11^. In the GCCS cohort, the *preSR* prevalence of SJL > 20 min is 82%, which is reduced *inSR* to 65%.

Unlike for the *preSR-inSR* associations of both SJL and sleep duration, the slope of regression line for mid-point of sleep on free days (MSF_sc_—chronotype) is close to 1 (at least within the 95-percentile range; Fig. 1b2). The fact that chronotype shows so much less “rubber band” effect underlines the stability of this phenotype. Nevertheless, on average, all chronotypes delay by about half an hour from *preSR* to *inSR*. This delay is more or less independent of *preSR* chronotype, which indicates that participants being exposed to less outside daylight inSR cannot simply explain the changes that we found. Responses to this zeitgeber change should be chronotype-specific^17, 41, 42^. This delay also cannot simply be explained by less morning-light exposure due to getting up later: *preSR* people (using alarm clocks on workdays) are exposed at different internal times to morning light—early chronotypes are exposed at a later internal phase than late chronotypes. Thus, late types will advance more than early types under strong STP and should respond stronger, i.e., chronotype-specific, when STP weakens *inSR*. When segregating the data into age-groups, the results do indicate a chronotype-specific response: the *preSR-inSR* slope in the younger participants, who are also the latest chronotypes within a population, deviates from 1 (see k-values in Fig. 2b). However, in this case, early types delay more than the late types. Since everybody delays and less STP is exerted in the mornings, it is likely that the early types comply with the increasing social pressure in the evenings (presumably via socializing in digital environments^43^ or with the family) and stay up later than they would under *preSR* conditions. This hypothesis would predict that early types get less sleep *inSR* than *preSR*, which we didn’t find. Another explanation would be that early chronotypes differ in sleep pressure buildup^44^ and therefore go to bed later *inSR* since they get more sleep.

SJL is predominantly caused by using an alarm clock to wake up on workdays^11, 45^, and our findings indicate that alarm clocks are circadian disruptors rather than synchronizers (supporting entrainment): alarm-clock use reduces the benefits for sleep duration and SJL associated with the lower STP *inSR*. In general, people approach their *preSR* free-day behavior even on *inSR* workdays, indicating that their free-day sleep without an alarm clock is a good surrogate for their actual circadian sleep window. This finding has important consequences. For example, decreasing sleep timing variability should be accomplished by changing work-day sleep timing rather than using alarm clocks also on free days; in other words, sleep on workdays and free days should be aligned by time-in-bed (onset) and not wake-up time (forced offset) as often suggested in the Internet (e.g., (https://www.youtube.com/watch?v=VzM459aKAJQ.)), but also in scientific publications^46^. The longer sleep on weekends or other work-free days fosters health and longevity^47^.

Chronotyping, assessment of sleep and circadian misalignment under conditions of quarantine provided insights to the actual *sleep need* of different age-groups and to the *tolerable SJL.* Our results indicate that long-term relaxation in STP should benefit health via increase in sleep duration, decrease in SJL and decrease in alarm-clock use. We conclude that alarm clocks are circadian disruptors, not synchronizers, and that the ubiquitous use of alarm clocks should be discouraged. Several large companies have already indicated to partly continue with work-from-home due to the positive experience under the recent social restrictions, and our data suggests that these reforms should benefit health. Altogether, the data suggest that current structuring of social schedules and changing our attitudes towards alarm clock use deserve serious consideration.

To the best of our knowledge, the GCCS is the largest published epidemiological study on changes in temporal behavior, including sleep, in the context of the COVID-19 pandemic, providing important insights. Current results may help in understanding of the consequences of social restrictions in the general population.

## Methods

### The GCCS

The internet-based Global Chrono Corona Survey (GCCS) study was approved by the Ariel University Human Research Ethics Committee of the Faculty of Health Sciences (AU-HEA-MK-20200629). All methods were performed in accordance with the relevant guidelines and regulations. All respondents were informed of the study purposes and of the names of the principal and coordinating investigators and provided electronic consent prior to commencement. Participation in the survey was anonymous; no personal identification data was requested.

We collected the data via SoGoSurvey platform (Herndon, Virginia, US), a cloud-based platform that enables creation and distribution of multilingual surveys. To translate the GCCS and advertise it world-wide, we formed an international network of colleagues and universities (see Acknowledgements). Recruitment methodologies included digital advertisements at universities, academic and non-academic social networks, and email-based approaches.

The GCCS contained 40–54 items, implemented in a smart-logic design where the presentation of the downstream questions depended on the answer to the key question (e.g., participants who declared unemployment, did not receive questions about habits on workdays). Participants could choose a preferred language (English, German, Hebrew, Arabic, Hindi, Japanese, Italian, Portuguese, Russian, and Spanish). Respondents were asked to provide basic demographic information (country, postal code, age, sex, height, weight, having/not having COVID-19 or history of COVID-19) and then to answer questions about daily behavior and lifestyle in separate sections, each pertaining to before social restrictions (preSocialRestriction, *preSR*) and during the restrictions (inSocialRestriction, *inSR*). Each section included questions about current work/study status, whether the work is a work-from-home or/and shift/night work. The core GCCS questions were about the sleep onset and wake-up times on workdays and on work-free days, typical meal times (not analyzed for this report) and exposure to outdoor daylight. Participants were asked about habitual use of alarm clock on workdays, and the time they spent outdoors in daylight on work- and work-free days. To avoid misunderstandings, participants were explicitly and repeatedly reminded to use the 24-h opposed to an AM-PM time-format. In the *inSR* section, additional questions queried for changes in subjective sleep quality, napping habits, quality of life, physical activity, time in front of screens and productivity, compared to the *preSR* period, using Likert scales (very negative (− 2), negative (− 1), no change (0), positive (+ 1), or very positive (+ 2) changes) for each wellbeing parameter.

### Study participants

11,431 respondents (≥ 18 years) from 40 countries completed the GCCS between April 4 and May 16, 2020; top response rates (> 200 respondents) came from Portugal, Italy, US, UK, Germany, Israel, India, Russia, Japan and Brazil. Exclusions for this analysis were responders diagnosed with COVID-19 (1.1%), shift/night workers (*preSR* or *inSR*, 16.3%), responders with extreme sleep durations (< 3 h and > 14 h; 3.1%), and those with missing or invalid data (8.3%). Occasional input errors due to confusion between time formats were systematically traced and manually checked. Obvious time format confusion cases were manually corrected, ambiguous cases were classified as invalid. The same researcher (M.K.) corrected/disqualified the cases in question, applying consistent criterions for decision-making. To correct for the overrepresentation of young (18–22y.o) participants from Russia relative to two other leading countries in this age group (India and Japan), we excluded 656 participants from Russia (5.7%) using random procedures.

The final sample included 7517 respondents (68.2% females). Length of stay *inSR* was calculated for each participant based on the response date and the onset date of the country-specific official social restrictions. Participants were on average 32.7 ± 9.1 days (range 10–59 days) under social restrictions or stay-at-home orders. We assumed this time duration allowed full adaptation to the new conditions and a steady-state for circadian entrainment phase. 80% of respondents worked or studied both *preSR* and *inSR* (66% from home). Full sociodemographic details are presented in Supplementary Material (SI-Table S1).

### Outcome measures

The following daily behaviors were calculated for each individual: (i) mean sleep duration across the week (SD_week_) was calculated as weighted average of the sleep duration on workdays (SD_W_, assuming 5 workdays) and work-free days (SD_F_), [SD_week_ = (5 × SD_W_ + 2 × SD_F_)/7]. (ii) Chronotypes (MSF_sc_), quantifying individual sleep timing as an indicator for phase of entrainment^11^. MSF_sc_ is based on the mid-point of sleep on free days (MSF) and corrected for sleep deficit accumulated on workdays. The mid-point of sleep was also calculated for workdays (MSW). (iii) As an assessment for circadian misalignment, social jet-lag (SJL) was calculated as the difference between MSF and MSW. To quantify the magnitude of changes in daily behavior we calculated individual restriction-induced Deltas (**∆**) as [*inSR*-*preSR*] for each parameter. The means and standard deviations of the main outcome parameters by country are reported in the SI-Table S5.

### Statistical analysis

We used non-parametric data analyses since none of the daily behavior variables showed normal distribution and/or was homoscedastic. Wilcoxon Matched-Pairs tests were used to assess the within-subject changes in daily behavior, effect sizes were calculated according to r = Z/√N^48^. Spearman’s rank correlation analysis was performed to assess associations between daily behavior measures, and light data. Mann–Whitney U-tests, with Glass rank-biserial correlation as the measure of effect size (r_g_ = 2(M1 − M2)/N1 + N2, where M1, M2—mean ranks, N1, N2—group sizes), were used to compare between the ad-hoc groups (e.g., age groups). LOESS regressions (locally weighted scatterplot smoothed curves)^49^ were applied on each parameter to smoothen it and to predict the Y (*inSR*) locally as a function of X (*preSR*) using the *stats* package (version 4.00) and plotting function *ggplot2* (version 3.3.2) in R. The size of the neighborhood was controlled using the span argument of 1. Statistical analyses were performed using SPSS version 26 (SPSS Inc., Chicago, IL, USA) and R; the level of significance was set at p < 0.05. Where appropriate, the p-value thresholds were adjusted for multiple comparisons.

### Study limitations

We did not obtain many relevant variables that may impact daily behaviors and also affect the response to social restrictions, such as general health, marital status, small children, socio-economic status, living in apartment *vs*. private house and many other. Other relevant variables, such as geographical factors (latitude and position in the time zone), dietary habits, exposure to daylight and wellbeing parameters were obtained but were not included in this report since it would have gone beyond the already complex scope of this paper. We note that the assessment of *preSR* parameters is based on retrospective reports and that only 32% percent of the sample were males. A possible validation to the retrospective sampling may come from comparison between current findings and a similar study by Write et al.^34^. In this study 139 university students from the University of Colorado Boulder (US), enrolled in the study at two time-points: at baseline, *preSR*, data collection was conducted as part of a class project between January 29 to February 4, 2020, while the *inSR* data was collected remotely between April 22 to April 29, 2020. The main findings of the two studies are in total accordance with each other, supporting the validity of the GCCS *preSR* data. We also acknowledge possible selection bias—we estimate that GCCS sample was drawn predominantly from people close to the academic circles, e.g., undergraduate and graduate students, university staff, biotech and high-tech workers and their family and social circles. However, the large sample size, geographical and cultural diversity, combined with the uniformity in the time spent under strict social restrictions (first wave COVID-19 responses), reduce any potential systematic bias.

## Supplementary Information

Supplementary Information.

## Data Availability

We included all the data needed for the evaluation of the conclusions in the Results section or in the Supplementary Information file. Additional data related to this article may be requested from the authors.
