# Supplementary material for: COVID-19-mandated social restrictions unveil the impact of social time pressure on sleep and body clock
Source: Sci Rep. 2020 Dec 17;10:22225. doi: 10.1038/s41598-020-79299-7 (PMC7746700; doi:10.1038/s41598-020-79299-7)
Supplement: Supplementary file 1 — Supplementary Information. [file 41598_2020_79299_MOESM1_ESM.pdf]

Supplementary Information: COVID-19-mandated social restrictions unveil the impact of social time pressure on sleep and body clock

Maria Korman, Vadim Tkachev, Cátia Reis, Yoko Komada, Shingo Kitamura, Denis Gubin, Vinod Kumar, Till Roenneberg

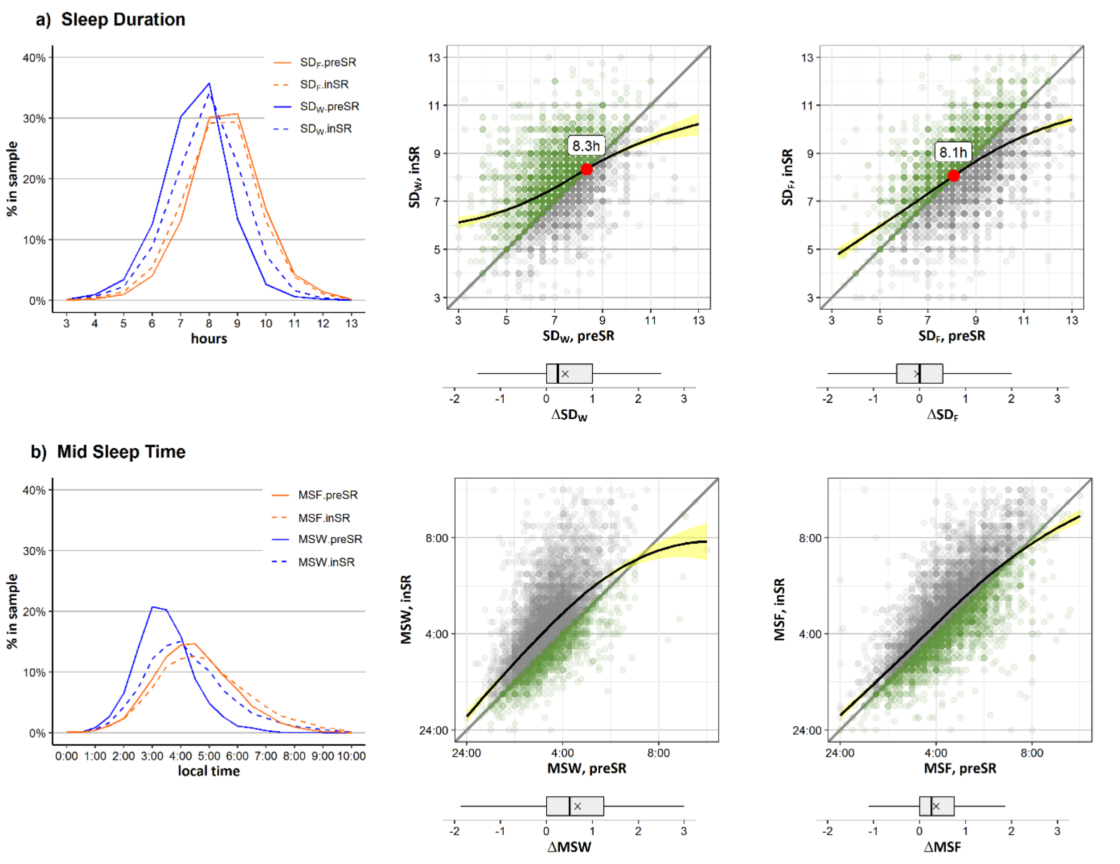

SI-Figure 1. Social restriction-induced changes (*preSR* -> *inSR*) in sleep-wake behavior in the general sample. **a) Sleep Duration** (hours) on workdays (SD_W) and free days (SD_F); and **b) Mid-Sleep time** (local time) on work (MSW) and work-free (MSF). For each parameter, distribution in the sample (smooth line – *preSR*; dashed line – *inSR*, blue - SD_W/MSW, orange – SD_F/MSF), scatterplots of individual values and boxplots of individual differences (Δ, hours) are presented. Overlapping dots are coded by color intensity. Diagonal line in the scatter plots designates no restriction-induced change in parameter. Green – increase/delay, grey – decrease/advance. Black LOESS regression lines illustrate the relationship between the parameter values *preSR* (x-axis) and the smoothed parameter values *inSR* (y-axis), pointwise 95%-confidence intervals are visualized by bands shaded in yellow. Red dot – intersection point between the diagonal and the LOESS line. In the boxplots: positive values – increase in SD_W/SD_F, delay in MSW/MSF; whiskers - max and min values, box borders – 75th and 25th percentiles, line through the box – median, × marker - mean.

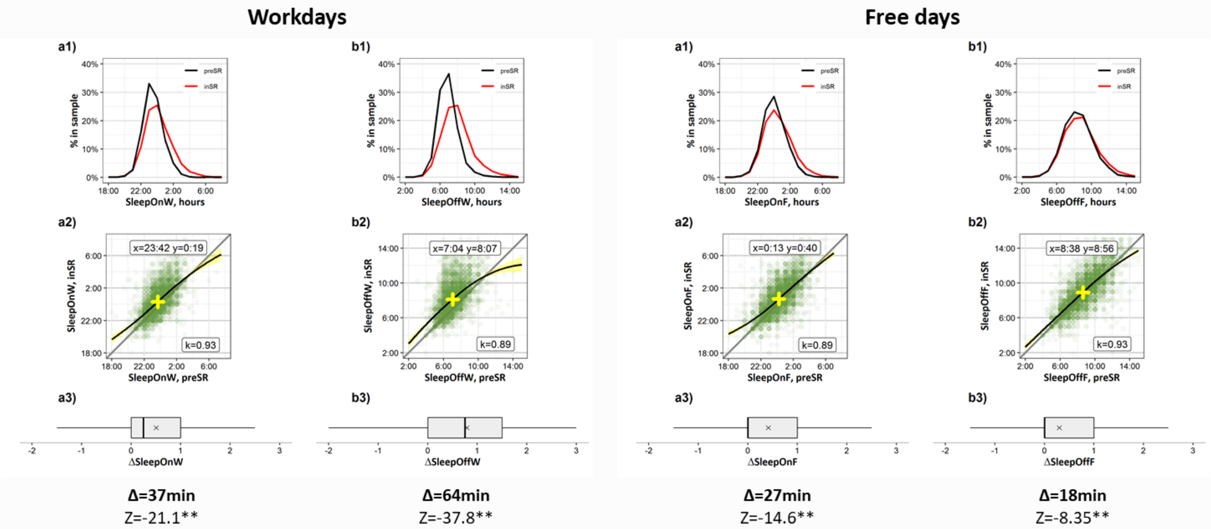

SI-Figure 2. Social restriction-induced changes (*preSR* -> *inSR*) in sleep onset and sleep offset times on workdays and free days (local time). **Distribution in the sample** (black line – *preSR*; red line – *inSR*): **a1)** Sleep onset on workdays/free days – SleepOnW/SleepOnF; **b1)** Sleep offset on workdays/free days – SleepOffW/SleepOffF. **Scatterplots of individual values**: **a2)** Sleep onset on workdays/free days – SleepOnW/SleepOnW; **b2)** Sleep offset on workdays/free days – SleepOffW/SleepOffW. Black LOESS regression lines illustrate the relationship between the parameter values *preSR* (x-axis) and the smoothed parameter values *inSR* (y-axis), pointwise 95%-confidence intervals are visualized by bands shaded in yellow. Yellow cross coordinates – mean of MSF_{sc} *preSR* (x-axis) and *inSR* (y-axis). k – slope of the tangent for the LOESS regression line at the point of mean value. **Boxplots of individual differences** (Δ, hours): **a3)** ΔSleepOnW/ΔSleepOnF; **b3)** ΔSleepOffW/ΔSleepOffW. Positive values – delay in onset or offset times; whiskers - max and min values, box borders – 75th and 25th percentiles, line through the box – median, × marker - mean. Mean Δ in minutes and Z statistic of the Wilcoxon Signed Ranks two-tailed tests (*preSR* -> *inSR*) for each parameter are shown below the boxplots. **p < 0.001.

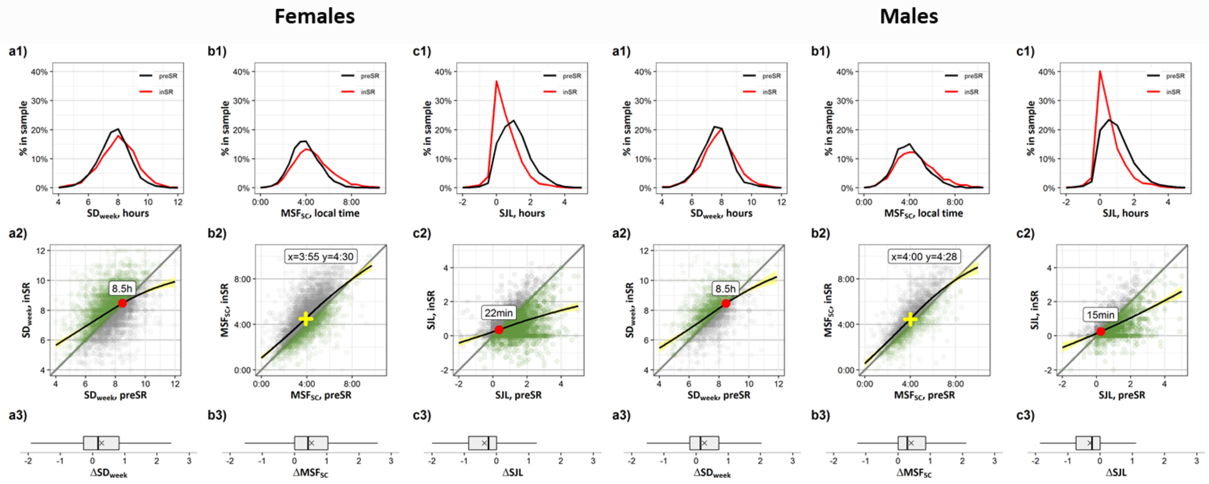

	Sex	N	Mean Rank	Mean Value	Mann-Whitney U	Z	r_g
**ΔSJL**	Female	4048	2962.59	31.5±56 min	3797373.0	-3.747, p<0.001	-0.06
	Male	1994	3141.10	24.8±54 min			
**ΔMSF_{SC}**	Female	5116	3841.68	36.7±74 min	5672746.0	-5.099, p<0.001	-0.07
	Male	2392	3568.05	28.9±72 min			
**ΔSD_{week}**	Female	5116	3761.69	15.4±69 min	6081947.0	-0.421, p - ns	-
	Male	2392	3739.12	15.1±61 min			

SI-Figure 3. Social restriction-induced changes (*preSR -> inSR*) in sleep-wake behavior by sex, females comprise 68.2% of the study sample. **Panels a1-c1 - Distributions of SD_{week} (hours), MSF_{sc} (chronotype, local time), and SJL (h),** *preSR* (black line) and *inSR* (red line), percent from group (Females or Males) total. **Panels a2-c2 - Scatterplots of SD_{week} (hours), MSF_{sc} (chronotype, local time), and SJL (h),** *preSR* (x axis) vs. *inSR* (y axis). Each dot represents an individual participant, overlapping dots are coded by color intensity. Diagonal line designates no restriction-induced change in parameter. Green – increase/advance, grey – decrease/delay. Black LOESS regression lines illustrate the relationship between the parameter values *preSR* (x-axis) and the smoothed parameter values *inSR* (y-axis), pointwise 95%-confidence intervals are visualized by bands shaded in yellow. Red dot – intersection point between the diagonal and the LOESS line. Yellow cross coordinates – mean of MSF_{sc} *preSR* (x-axis) and *inSR* (y-axis). **Panels a3-a3 – Boxplots of individual differences (Δ, hours) in SD_{week}, MSF_{sc} and SJL**. Positive values – increase in SD_{week}, delay in MSF_{sc}. Negative values - decrease in SJL. Whiskers - max and min values, box borders – 75th and 25th percentiles, line through the box – median, × marker - mean. **Table** - Mann–Whitney U-test statistics of group comparisons. No differences between sex groups in ΔSD_{week} were found; sex groups had minor but significant differences in ΔMSF_{sc} (7.8 min, females have larger delay) and ΔSJL (6.7 min, females have larger shift), with negligible effect sizes (r_g < 0.1).

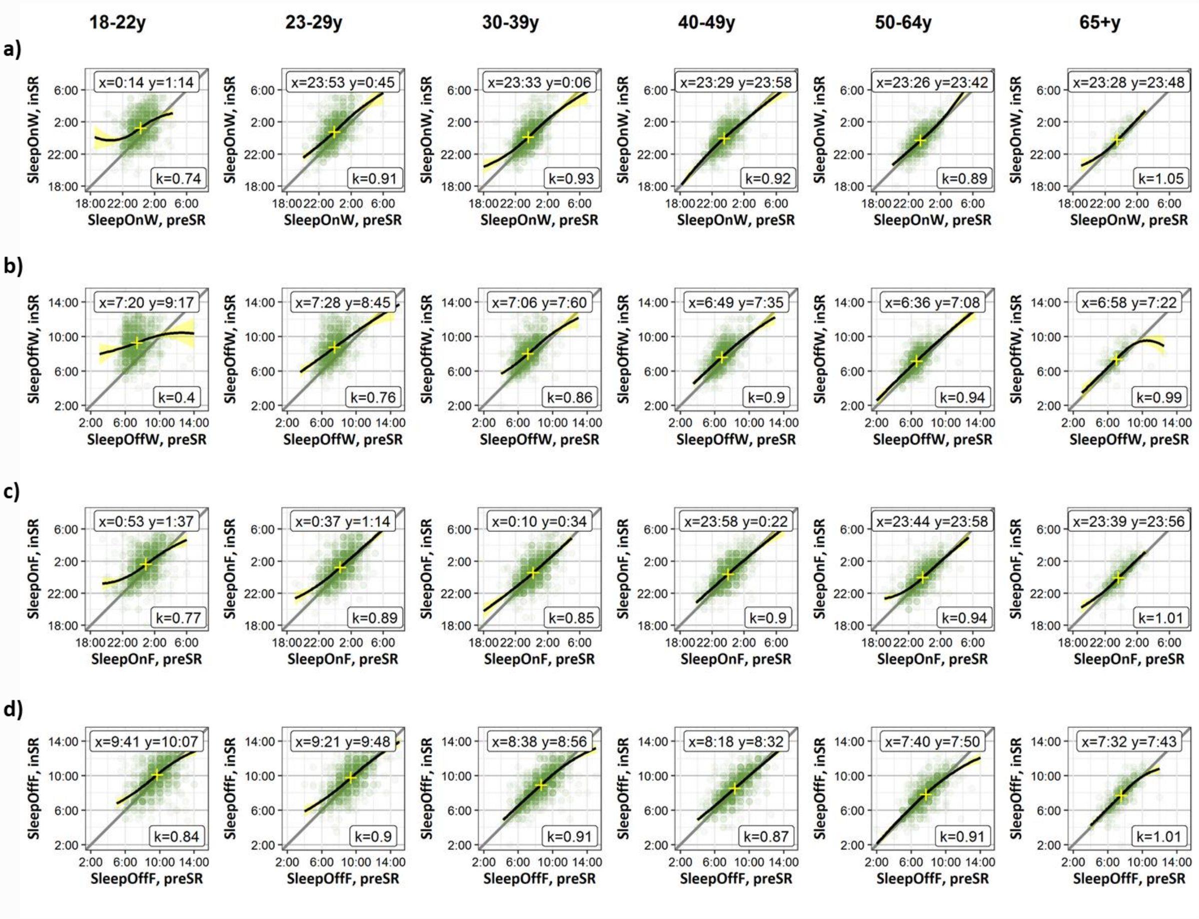

SI-Figure 4. Social restriction-induced changes (*preSR* -> *inSR*) in sleep onset and sleep offset on work and work-free days by age-group (18-22y, 23-29y, 30-39y, 40-49y, 50-64y, and 65+y): **a) Sleep onset on workdays** – SleepOnW; **b) Sleep offset on workdays** - SleepOffW; **c) Sleep onset on free days** – SleepOnF; **d) Sleep offset on free days** - SleepOffF. Black LOESS regression lines illustrate the relationship between the parameter values *preSR* (x-axis, local time) and the smoothed parameter values *inSR* (y-axis, local time), pointwise 95%-confidence intervals are visualized by bands shaded in yellow. Yellow cross coordinates – mean of parameter *preSR* (x-axis) and *inSR* (y-axis). k – slope of the tangent for the LOESS regression line at the point of mean parameter value *preSR.*

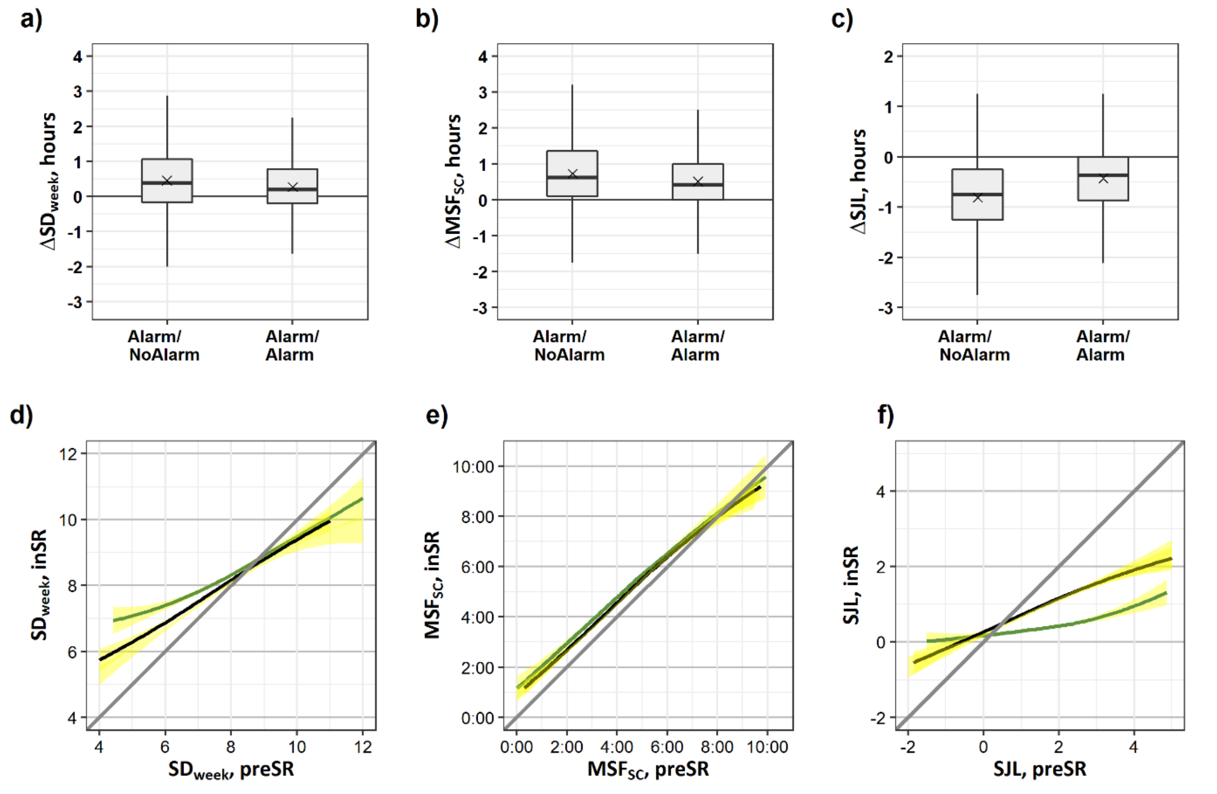

SI-Figure 5. The contribution of alarm clock use on work on changes in daily behavior in Alarm/NoAlarm and Alarm/Alarm groups. **Upper panel.** Boxplots of individual differences (Δ, hours) in sleep-wake parameters according to alarm clock use inSR: Alarm/NoAlarm (N=1723) and Alarm/Alarm (N=3337) groups**. a)** SD_{week}, positive values – increase**; b**) MSF_{sc}**,** positive values – delay and **c)** SJL, positive values – increase**,** *preSR* and *inSR.* Note differences in scale and different number of participants in the groups. Whiskers - max and min values, box borders – 75th and 25th percentiles, line through the box – median, × marker - mean. **Lower panel.** Black LOESS regression lines by group, Alarm/NoAlarm (green lines) and Alarm/Alarm (black lines), **d)** SD_{week} (hours), **e)** MSF_{sc} (chronotype, local time), and **f)** SJL (hours), *preSR* (x axis) vs. *inSR* (y axis). Note that in alarm and no-alarm clock users, the intersection points are roughly the same, suggesting that the differences obtained between the groups are due to use of alarm clock.

SI Table 1. *Sociodemographic details,* **General sample**

**N**	**7517**
**Sex**	
Male	32%
Female	68%
**Age**	
Mean	38.5
SD	14.8
**Age Group**	
18-22y	16%
23-29y	18%
30-39y	22%
40-49y	20%
50-64y	17%
65+y	6%
**Alarm clock usage on workdays**	
preSR	77%
inSR	45%
**Employed/Student**	
preSR	92%
inSR	81%
**Work/Study from home**	
preSR	11%
inSR	66%
**Days from social restrictions onset**	
Mean	32.7
SD	9.1

Top countries (N)

Russia	1089
Japan	1080
Portugal	860
India	766
US	729
Italy	628
UK	417
Israel	341
Germany	320
Brazil	142

Distribution of GCCS respondents. The heat map was generated in Excel (Microsoft Office 10) using Bing Map app according to the geographical data reported by the participants (country and ZIP code).

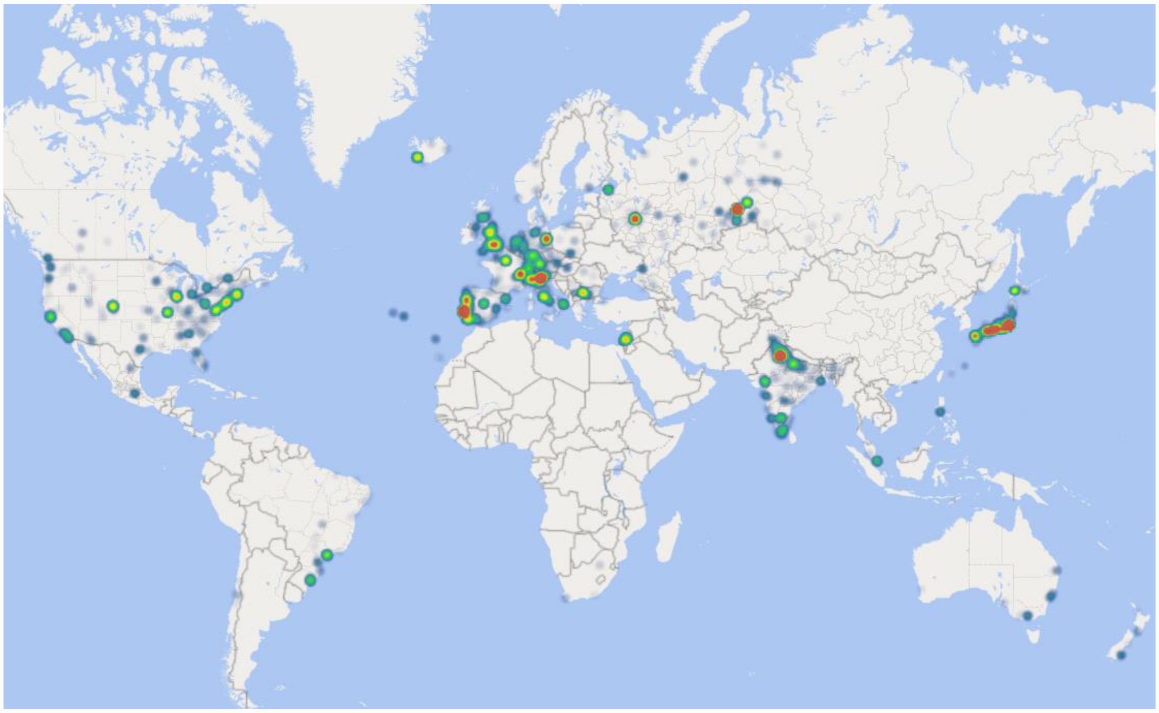

SI Table 2. *Sociodemographic details,* **Age groups**

	**18-22y**	**23-29y**	**30-39y**	**40-49y**	**50-64y**	**65+y**
**N**	1225	1340	1660	1515	1312	456
**Sex**						
**Male**	25%	30%	33%	32%	35%	45%
**Female**	75%	70%	67%	68%	65%	55%
**Age (years)**						
**Mean**	20	25.8	34.3	44.3	56.1	70.4
**SD**	1.4	2	2.9	2.9	4.2	4.8
**Mean MSF_{sc} (chronotype, local time)**						
**preSR**	4:39	4:33	4:01	3:46	3:26	3:30
**inSR**	5:39	5:16	4:31	4:14	3:44	3:46
**Alarm clock usage on workdays**						
**preSR**	89%	86%	80%	81%	66%	23%
**inSR**	42%	53%	50%	50%	41%	12%
**Employed/Student**						
**preSR**	99%	97%	93%	95%	89%	48%
**inSR**	86%	87%	83%	84%	79%	42%

	18-22y	23-29y	30-39y	40-49y	50-64y	65+y
**Ratio (Alarm clock usage / Employed)**						
**preSR**	0.90	0.89	0.86	0.85	0.74	0.48
**inSR**	0.49	0.61	0.60	0.60	0.52	0.29
**Work/Study from home**						
**preSR**	12%	10%	10%	11%	11%	16%
**inSR**	70%	75%	71%	66%	60%	32%
**Days from social restrictions onset**						
**Mean**	34.4	33.2	32.4	31.4	32.1	34.1
**SD**	7	8.8	9.6	9.5	9.6	10.2
**Top countries (N)**						
	Russia 350	India 204	Portugal 213	Portugal 275	Japan 249	US 106
	India 281	US 182	Russia 210	Russia 249	US 158	Italy 105
	Japan 260	Russia 138	Japan 194	Japan 220	Italy 155	Japan 39
	Portugal 67	Portugal 122	US 135	US 110	Portugal 153	Israel 37
	Italy 65	Japan 118	India 130	UK 92	Russia 108	Russia 34
	UK 43	Italy 91	Italy 123	Italy 89	UK 83	Portugal 30

SI Table 3. *Sociodemographic details,* ***Alarm/NoAlarm*** *vs****. Alarm/Alarm group***

	Alarm/NoAlarm	Alarm/Alarm
**N**	1539	2596
**Sex**		
Male	31%	31%
Female	69%	69%
**Age (years)**		
Mean	35	35.2
SD	13.2	12.2
**Age Group**		
18-22y	23%	16%
23-29y	20%	23%
30-39y	22%	25%
40-49y	19%	21%
50-64y	14%	13%
65+y	2%	1%
**Alarm clock usage on workdays**		
preSR	100%	100%
inSR	0%	100%
**Employed/Student**		
preSR	100%	100%
inSR	100%	100%
**Work/Study from home**		
preSR	12%	12%
inSR	100%	100%
**Days from social restrictions onset**		
Mean	32.4	33.1

SD	8.3	9.5
**Top countries (N)**		
	Russia 291	Portugal 409
	India 210	Japan 359
	Portugal 170	Russia 315
	US 157	US 290
	Japan 125	Italy 222
	UK 105	UK 166
	Italy 71	Germany 125
	Germany 67	India 125
	Israel 44	Israel 72
	Switzerland 40	Switzerland 68

SI Table 4. *Comparisons between the deltas* ***[inSR-preSR]*** *of the main outcome measures of daily behaviors (*ΔSD_{week}*,* ΔMSF_{sc} *and* ΔSJL*) in the three ad-hoc groups: people* ***working from home*** *(N = 4977, mean age 36.5+/-13.5 years), those that* ***worked not from home*** *(N = 1126, mean age 39.7+/-14.5 years), and those that* ***did not work*** *(N = 1414, mean age 44.5+/-17.6) inSR. Means of individual deltas ± sd (hh:mm)***,** *Z statistic and p-value of the Mann-Whitney U tests for each parameter are shown (not corrected for the multiple comparisons). Note that there were significant differences in the mean age of the groups, this fact may bias the interpretation of the pairwise comparisons between the groups.*

	**Work from home**	**Work not from home**	**Z**	**p-value**
ΔSD_{week}	0:17+/-1:02	0:12+/-1:02	3.15	0.002
ΔMSF_{sc}	0:34+/-1:10	0:20+/-1:07	10.7	< 0.001
**ΔSJL**	-1:28+/-0:55	-1:44+/-0:56	-11.7	< 0.001
**age**	36.5+/-13.5	39.7+/-14.5	-6.72	< 0.001

	**Work from home**	**Don't work**	**Z**	**p-value**
ΔSD_{week}	0:17+/-1:02	0:11+/-1:21	3.74	< 0.001
ΔMSF_{sc}	0:34+/-1:10	0:45+/-1:27	-2.9	0.004
**ΔSJL**	-1:28+/-0:55	-	-	-
**age**	36.5+/-13.5	44.5+/-17.6	-14.7	< 0.001

	**Work not from home**	**Don't work**	**Z**	**p-value**
ΔSD_{week}	0:12+/-1:02	0:11+/-1:21	0.879	0.379
ΔMSF_{sc}	0:20+/-1:07	0:45+/-1:27	-10.2	< 0.001
**ΔSJL**	-1:44+/-0:56	-	-	-
**age**	39.7+/-14.5	44.5+/-17.6	-6.34	< 0.001

SI Table 5. *Main outcome measures of daily behaviors (mean ± sd) in six top countries of the GCCS sample* ***preSR*** *and* ***inSR****: sleep duration,* SD_{week}*, corrected mid-sleep time on free days,* MSF_{sc} *(chronotype), and social jetlag, SJL. Z statistic and p-value of the Wilcoxon Signed Ranks two-tailed tests (preSR -> inSR) and ∆* ***[inSR-preSR]*** *for each parameter are shown.*

	preSR	***inSR***	**Z**	**p-value**	**∆ [*inSR-preSR*]**
**Russia N = 1089**					
SD_{week}	7:40+/-1:09	8:21+/-1:18	-13.5	< 0.001	0:41+/-1:21
MSF_{sc}	4:15+/-1:24	5:07+/-1:56	-11.3	< 0.001	0:52+/-1:25
SJL	1:29+/-1:01	0:33+/-0:52	20.6	< 0.001	-1:03+/-1:09
**Japan N = 1080**					
SD_{week}	6:58+/-1:03	7:12+/-1:12	-4.59	< 0.001	0:13+/-0:57
MSF_{sc}	4:01+/-1:25	4:17+/-1:54	-2.35	0.018	0:15+/-1:09
SJL	0:55+/-0:55	0:39+/-0:50	8.16	< 0.001	-1:44+/-0:51
**Portugal N = 860**					
SD_{week}	7:49+/-0:55	7:59+/-1:10	-4.2	< 0.001	0:10+/-1:02
MSF_{sc}	4:26+/-1:08	5:08+/-1:24	-10.8	< 0.001	0:41+/-1:01
SJL	1:13+/-0:50	0:47+/-0:44	11.1	< 0.001	-1:33+/-0:47
**India N = 766**					
SD_{week}	7:41+/-1:12	7:50+/-1:17	-2.03	0.042	0:08+/-1:19
MSF_{sc}	3:46+/-1:39	4:35+/-2:01	-7.74	< 0.001	0:48+/-1:37
SJL	0:48+/-1:04	0:13+/-0:42	13.2	< 0.001	-1:26+/-1:06
**US N = 729**					
SD_{week}	8:05+/-0:57	8:16+/-1:09	-3.4	< 0.001	0:10+/-0:59
MSF_{sc}	3:38+/-1:27	4:09+/-1:46	-5.53	< 0.001	0:31+/-1:04
SJL	0:59+/-0:53	0:38+/-0:46	8.09	< 0.001	-1:39+/-0:52
**Italy N = 628**					
SD_{week}	7:43+/-0:56	7:51+/-1:05	-3.05	0.002	0:07+/-0:58
MSF_{sc}	4:07+/-1:13	4:23+/-1:21	-3.85	< 0.001	0:16+/-0:52
SJL	1:10+/-0:55	0:38+/-0:43	10.2	< 0.001	-1:29+/-0:46
